# Neurologic Complications Associated with Sjögren's Disease: Case Reports and Modern Pathogenic Dilemma

**DOI:** 10.1155/2014/590292

**Published:** 2014-08-05

**Authors:** Michele Colaci, Giulia Cassone, Andreina Manfredi, Marco Sebastiani, Dilia Giuggioli, Clodoveo Ferri

**Affiliations:** Chair and Rheumatology Unit, Medical School, Azienda Ospedaliero-Universitaria, University of Modena and Reggio Emilia, Policlinico di Modena, Via del Pozzo 71, 41100 Modena, Italy

## Abstract

*Objectives*. Sjögren's syndrome (SS) may be complicated by some neurological manifestations, generally sensory polyneuropathy. Furthermore, involvement of cranial nerves was described as rare complications of SS. *Methods*. We reported 2 cases: the first one was a 40-year-old woman who developed neuritis of the left optic nerve as presenting symptom few years before the diagnosis of SS; the second was a 54-year-old woman who presented a paralysis of the right phrenic nerve 7 years after the SS onset. An exhaustive review of the literature on patients with cranial or phrenic nerve involvements was also carried out. *Results*. To the best of our knowledge, our second case represents the first observation of SS-associated phrenic nerve mononeuritis, while optic neuritis represents the most frequent cranial nerve involvement detectable in this connective tissue disease. Trigeminal neuropathy is also frequently reported, whereas neuritis involving the other cranial nerves is quite rare. *Conclusions*. Cranial nerve injury is a harmful complication of SS, even if less commonly recorded compared to peripheral neuropathy. Neurological manifestations may precede the clinical onset of SS; therefore, in patients with apparently isolated cranial nerve involvement, a correct diagnosis of the underlying SS is often delayed or overlooked entirely; in these instances, standard clinicoserological assessment is recommendable.

## 1. Introduction

Sjögren's syndrome (SS) is characterized by chronic inflammation of exocrine glands, such as lachrymal and salivary glands, leading to xerophthalmia and xerostomia [1]. Moreover, at least one third of patients present with systemic extraglandular neurological, articular, pulmonary, or gastrointestinal manifestations [2]. The prevalence of neurological manifestations in SS varies between 2% and 60%, mostly pure or predominantly sensory polyneuropathies [3]; therefore, a careful neurological evaluation is usually recommended in the global assessment of SS patients [4]. Apart from central nervous system (CNS) involvement, other neuropathic patterns are polyradiculopathies, mononeuritis multiplex, autonomic neuropathies, and cranial neuropathies [5]. These latter are not rarely observed in SS patients, often as presenting symptom in the absence of overt sicca syndrome [6, 7], thus the differential diagnosis may be quite difficult.

The pathogenic mechanisms responsible for SS neurological involvement are unknown. Many hypotheses have been considered, namely, the direct infiltration of the central nervous tissue by lymphocytic cells, the vascular injury due to antineuronal and anti-SSA antibodies, and/or the ischemia secondary to small vessel vasculitis [8].

In the present paper, we described two patients with SS, who presented mononeuritis: the first patient presented optic neuritis preceding the clinical onset of SS and the second developed a very unusual neuritis of the right phrenic nerve. Finally, a review of the literature on cervicocranial neuritis in the course of SS was carried out.

## 2. Case Reports

### 2.1. Case 1

A 40-year-old woman referred to our Rheumatology Unit in January 2012, in the suspicion of an autoimmune disorder. Since 2006, she presented episodes of left optic mononeuritis, responsive to steroids; thus, she was undergoing a neurologic follow-up for a hypothetical multiple sclerosis. The first magnetic resonance (MR) of the brain and the spine showed a solitary T2-hyperintense, gadolinium enhancing lesion of the retrobulbar and intracanalicular left optic nerve, suggestive for demyelinated plaque; anyway, the subsequent yearly MRs did not show other lesions. Since 2006, positivity for antinuclear antibodies (ANA) at the titre of 1 : 640 (speckled) was detected, along with reduced lymphocyte counts (500–600 cells/*μ*L out of 3,500–4,000 total white cells); the screening for HCV and HBV infections was negative. Furthermore, since 2007 polyclonal hypergammaglobulinemia, slight reduction of C3 fraction of complement, and serum antitransglutaminase and anti-SSA/SSB autoantibodies were found. However, the patient did not present a clinically overt sicca syndrome; thus SS remained misdiagnosed until 2012.

Only after the patient referred to our Rheumatology Centre, a deeper check-up was performed, namely, salivary glands scintigraphy that showed mild salivary hyposecretion basally and after lemon juice stimulation and Schirmer's test that was bilaterally positive (<5 mm). On these bases, a diagnosis of SS, classified according to the new American College of Rheumatology/Sjögren's International Collaborative Clinical Alliance (ACR/SICCA) criteria [9],* with recurrent, unilateral optic neuritis* was done. Antibodies targeting aquaporin-4 (NMO antibodies) were not tested for in the serum because the test was not available; anyway, the patient did not show acute myelitis or spinal cord MRI lesions. Moreover, the possibility of optic neuritis not SS-related, but incidental only, cannot be excluded.

No chronic treatment was prescribed, except steroids in the case of the further two episodes of mild optic neuritis.

### 2.2. Case 2

A 54-year-old woman with diagnosis of SS-scleroderma overlap syndrome from 2005 referred to our Rheumatology Unit in January 2013. She did not present skin involvement, while an overt sicca syndrome was observed. Serum anticentromere autoantibodies were present, without anti-ENA specificity; Schirmer's test was clearly positive (<1 mm). Videocapillaroscopy revealed an “early” scleroderma pattern; salivary glands echography evidenced a complete atrophy, while chest high-resolution computed tomography did not show interstitial lung disease, but only hypotonia of oesophagus. A labial salivary gland biopsy was not performed, since the patient refused it; thus, the diagnosis of primary SS, classified according to ACR/SICCA criteria [9], cannot be formulated.

Since 2012, the patient progressively complained of fatigue and dyspnoea in the absence of new radiological pulmonary alterations; therefore, oxygen therapy (3 L/min) was necessary. Forced vital capacity was 50% of normal value, whereas heart echography was normal. In November 2012, the diagnosis of paralysis of right diaphragm was suspected on the bases of clinical symptoms and radiological alterations at standard chest X-ray and then confirmed by the detection of mixed demyelinated/axonal damage of right phrenic nerve at electromyographic examination. A modest axonal polyneuropathy of the arms and the legs was also present, while neck MR excluded compressions of nerve roots. Treatment with steroids at 1 mg/kg/day was prescribed without significant improvement; thus the patient was referred to our Rheumatology Unit, where an immune-mediated mononeuritis of the right phrenic nerve was suspected. Therapy with monthly cycles of intravenous immunoglobulin (50 g/day, for 3 consecutive days) was decided. After the third cycle, the patient experienced reduction of dyspnoea (from 10/10 to 6/10 of modified Borg scale), improved physical performance, and tapering of daily oxygen therapy, without disease exacerbation up to date.

## 3. Review of the Literature

An exhaustive revision of the literature was done by including all case reports and case series of SS patients with cervical-cranial neuritis present in PubMed database (Table 1); reports regarding SS associated with other connective tissue diseases were excluded. Ninety-five reports with a total of 267 SS patients with different types of cranial neuritis during their clinical history were found [4, 7, 10–101]; moreover, 68 reports with available data referred to 160 patients were analyzed in detail. In particular, the female/male ratio was 20.8, while the mean age of adult SS patients is 48 ± 13.2 years, consistently with recent SS epidemiological findings [102, 103]. Four cases of young girls (range 8–11 years) were also reported, all affected by optic neuritis and two by CNS involvement [34, 45, 63, 67]. Of interest, considering the 75 cases with available data, the first episode of cranial neuritis was contemporary to SS diagnosis in 40% of patients, while neuritis proceeded in 24% (range 1–35 years), or followed SS diagnosis (range 1–14 years) in 36%, respectively.

### 3.1. Ipo-Anosmia (I Cranial Nerve)

Alterations of the I cranial nerve were rarely observed; to date, only 4 cases were described [14, 59]. However, it may be supposable that this complication is often underdiagnosed or misdiagnosed in SS patients because of the concomitant olfactory and gustatory dysfunctions secondary to mucosal dryness.

### 3.2. Optic Neuritis (II Cranial Nerve)

The review of the literature revealed that optic neuritis represents the 46.4% (124/267) of cranial neuritis in SS patients [7, 18, 19, 24, 34, 36, 43–45, 49, 54–56, 59, 63, 65–68, 70, 72–75, 77–81, 83, 85–93, 95–97, 100, 104]; it is often bilateral with contemporary [65, 67, 68, 70, 85, 89, 95, 97] or sequential [36, 63] occurrence. In a few cases, neuritis was associated with CNS involvement, such as brain vasculitis [34, 77], acute transverse myelopathy [43, 67], myelitis [77, 80, 83, 90], aseptic meningitis [70], or multiple sclerosis-like features [80, 91]. Response to treatment was frequently effective, especially with the use of immunosuppressors; however, an irreversible visual impairment was occasionally reported [45, 63, 79, 95]. From a pathophysiological point of view, optic neuropathy may be related to immune-mediated small vessel vasculitis [70]; otherwise, it may be due to demyelinating disease being included or not in the neuromyelitis optica spectrum disorder [91].

The majority of published papers refer to case reports. Among cohort studies, Alexander et al. [18] investigated the presence of CNS involvement in a cohort of more than 200 SS patients, referring to the John Hopkins Medical Institute between 1980 and 1983. The 20% of these patients showed CNS complications, including 16 cases of cranial nerve involvement. In particular, 7 patients presented transient monocular optic neuritis, with new episodes of neuritis of the other eye in 5 cases. Gono et al. in 2011 [91] reported CNS complications in 56/82 SS patients, 13 of them with optic neuropathy. The spectrum of clinical features was heterogeneous, but the majority of patients presented focal or multifocal alterations preceding the diagnosis of SS. Moreover, the authors underlined that these disease complications could mimic multiple sclerosis features with possible misdiagnoses; on the other hand, they stressed the importance of screening SS patients in presence of neurologic manifestations.

### 3.3. Diplopia (III-IV-VI Cranial Nerves)

Diplopia is one of the most evident features related to alterations of cranial nerves, namely, oculomotor, trochlear, and abducens. Specific involvement of the III nerve in SS patients was described in 9 cases [4, 11, 18, 38, 57, 64, 69, 71], of the VI in 7 cases [18, 62], while only one patient with IV nerve alteration is present in the literature [47]. Moreover, several other reports illustrated cases of multiple neuritis including diplopia among the clinical features [7, 13, 17, 36, 41, 42, 48, 50, 60, 82]. The prognosis is generally good, with complete resolution within few weeks after steroid treatment; however, possible recurrent episodes have been reported [36, 38, 41, 48].

### 3.4. Trigeminal Neuropathy (V Cranial Nerve)

Trigeminal injury is the second more frequently described feature among cranial neuritis in SS patients; in fact, trigeminal neuropathy was observed in 102/267 (38%) cases, isolated or associated with other cranial nerve involvement [7, 12, 14–18, 20–23, 25, 26, 29–33, 37, 39–42, 44, 46, 50, 51, 53, 58–60, 76, 82, 84, 87, 91, 94, 98, 99]. In 13 cases the neuritis was bilateral [12, 14, 31, 44, 60, 94], while in 2 it was combined to dorsal roots ganglionitis [21, 25]. The latter association may suggest a possible pathogenic mechanism of trigeminal injury that is an immune-mediated neuron death in the sensory Gasser ganglion [60]. The prominence of the sensory symptoms or the presence of several SS patients with pure sensitive trigeminal neuritis in literature may reinforce this hypothesis [14, 26, 31, 40, 46].

Considering large cohort studies, Mellgren et al. [29] retrospectively investigated the features of neural involvement in a cohort of 110 SS patients complicated by neuropathy, referred to Mayo Clinic between 1976 and 1988. Trigeminal neuropathy was recorded in 5 patients, generally associated with sensory motor polyneuropathy. In 2005, Mori et al. [60] described 92 Japanese patients with SS and neuropathy; to note, in 93% of cases the diagnosis of SS followed the onset of neural disorders. Pure sensory trigeminal neuritis affected 15 patients (6 with bilateral involvement), while 5 individuals showed multiple cranial neuropathy patterns. Overall, the prevalence of trigeminal neuropathy in SS patients seems to vary depending on ethnic factors. Anyway, the involvement of V cranial nerve in the course of SS is not rare; moreover, it tends to be recurrent or to stabilize and to be less frequently responsive to steroids than other cranial neuritis [101].

### 3.5. Facial Palsy (VII Cranial Nerve)

In 1935, Henrik Sjögren himself was the first author to mention cranial nerve involvement in SS patients, describing a case with bilateral facial palsy [9]; up to date, other 13 cases were described [18, 28, 35, 52, 59–61, 78, 86, 91], with bilateral involvement in only one patient [28]. In other cases, facial involvement presented as multiple cranial neuritis [7, 13, 17, 48, 82], usually associated with trigeminal and/or glossopharyngeal nerve injury, generally responsive to steroid treatment.

### 3.6. Tinnitus (VIII Cranial Nerve)

Neuropathy of the cochlear nerve is rarely reported in the literature. Except one [41], they were described as isolated involvement [4, 18, 59]. Significant data regarding prognosis were lacking.

### 3.7. Involvement of IX, X, XI, and XII Cranial Nerves

A few reports described SS patients who presented difficulty of swallowing, which is a symptom of glossopharyngeal neuropathy [13, 17, 36, 48, 51, 60, 76, 91]; this nerve involvement was always reported in cases of multineuritis. Also the vagus and the hypoglossal nerves were rarely mentioned in the literature and invariably associated with the IX cranial nerve [48, 51, 60, 76, 91]. All cases seem to be related to transient, often recurrent episodes of multineuritis, generally responsive to treatment. Finally, the XI nerve involvement was never reported.

### 3.8. Phrenic Nerve Involvement

Phrenic nerve is not a cranial nerve; it originates in the neck from C3–C5 cervical nerves, containing motor efferent fibres to diaphragm and sensitive fibres from pericardium and visceral pleura.

The research in PubMed database of “Phrenic nerve”* and* “Sjögren's syndrome” did not find any report. Furthermore, considering other autoimmune rheumatic diseases, few anecdotal reports regarding phrenic nerve involvement in systemic lupus erythematosus [105–108] or ANCA-associated vasculitides [109–112] were found. Up to date, diaphragm paralysis is very rarely associated with autoimmune disorders; therefore we cannot exclude that the present case number 2 represents a casual association with SS than SS-related.

## 4. Discussion

In the present report, two SS patients with optic and phrenic mononeuritis were described. The coexistence of a cranial neuritis in the course of SS is in keeping with the world literature reporting a number of anecdotal cases and a few cohort studies describing SS patients with cranial neuritis [4, 7, 10–101]; however, to the best of our knowledge, our observation of phrenic mononeuritis represents the first patient with SS complicated by such peculiar manifestation.

Involvement of cranial nerves in SS is much less common with respect to peripheral neuropathy [91]; however, cranial neuritis may be considered relatively frequent among central nervous system neuropathic patterns (Table 2). It is not always clear whether optic neuritis, a demyelinating disease characterized by significant morbidity in more than 90% of cases [95], is secondary or coincidental with SS; anyway, both SS and optic neuritis seem to be autoimmune-driven disorder [60, 91, 93, 97]. In this respect, neuromyelitis optica (NMO), also named Devic's disease, is a heterogeneous disease, characterized by chronic demyelinating inflammation of the optic nerve and the spinal cord, mostly characterized by the presence of antibodies targeting aquaporin-4 (NMO antibodies) with a specificity of 90% [113]; this autoantigen, expressed on astrocytes in a perivascular distribution, represents a water channel regulating the water homoeostasis in the central nervous system [114]. Initially considered as a localized form of multiple sclerosis, NMO is now classified as independent nosologic entity [115]; in 2006, modified diagnostic criteria were proposed [116], including the presence of optic neuritis, acute myelitis, and at least 2 out of the 3 following supportive criteria: contiguous spinal cord MRI lesion extending over ≥3 vertebral segments, brain MRI not meeting diagnostic criteria for multiple sclerosis, and NMO-IgG seropositive status. A few cases reported in the literature regarding SS patients with optic neuritis may be diagnosed as NMO [83, 90, 113]; indeed, many of the “SS optic neuritis” patients were described prior to the discovery of the NMO antibodies, and therefore many of these cases may actually represent NMO, and not SS-related. On the other hand, in the study by Wingerchuk et al. [104] among 71 subjects with NMO, 4 patients affected by SS were mentioned; therefore, we may assume that SS and NMO could represent comorbidity. Interestingly, the presence of NMO antibodies in SS patients has been described [75, 93, 95, 97], possibly as expression of polyclonal B cell activation typical of SS [95]; on the other hand, patients with NMO frequently have other autoantibodies of a variety of specificities, and among the commonest of these autoantibodies are SSA and SSB [74]. A comparable autoimmune mechanism against both protein sequences of aquaporin-4 (antigen specific for NMO) and aquaporin-5 (expressed in salivary glands) [113] could be hypothesized, even if evidences are still lacking. In these lights, a rheumatologic screening for latent SS is recommendable in patients with apparently isolated optic demyelinating lesions or already classified as NMO.

A pure sensory trigeminal neuropathy has been described in patients with SS [14, 26, 31, 40, 46]. Since the trigeminal sensory neurons conducing cutaneous stimuli are located in the Gasser ganglion, damage at this level is suspected. This hypothesis is supported by the study of Valls-Sole et al. [117], who carried out an electrophysiological study of the trigeminal-facial and trigeminal-trigeminal reflexes in 5 SS patients with pure sensory neuropathy, compared to subjects with sensory-motor neuropathies from other causes and to healthy controls. SS patients with pure sensory neuropathy specifically presented an abnormal blink reflex and a normal jaw jerk. These findings suggest that the damage involves the neurons of the Gasser ganglion and not the axons of the V cranial nerve.

In many cases described in literature, as well as in our patient number 1, SS was diagnosed contemporary or even after the onset of cranial neuritis. These patients are commonly referred to neurology or ophthalmology centres, where sicca syndrome or other SS manifestations, such as arthralgias and/or mild arthritis, may be underestimated or not even mentioned by the patients. Consequently, a correct diagnosis of the underlying connective tissue disease is often delayed or overlooked entirely. Nonetheless, patients with CNS involvement and/or optic neuritis may be misdiagnosed as multiple sclerosis. Thus, in patients with cranial nerve involvement it is recommendable to keep in mind the possibility of associated SS that may be easily diagnosed by standard clinicoserological assessment.

With regard to our patient number 2, the diagnosis of overlapping SS and systemic sclerosis was based on the presence of typical SS clinical manifestations, plus anticentromere autoantibodies, esophagus involvement, and scleroderma capillaroscopic pattern [118]. Yet, the typical anti-SSA/SSB autoantibodies were not found, nor a lip biopsy was carried out to ascertain the diagnosis of SS. Anyway, on the basis on the symptom complex described above, the patient might be included in the subset of patients with the so-called “ACA-positive limited scleroderma/SS overlap syndrome,” which is characterized by benign scleroderma clinical course but at a high risk of non-Hodgkin's lymphoma [119]. Interestingly, an increased frequency of peripheral neuropathy in this peculiar clinical subset has been previously reported [120]. In regard to the phrenic nerve involvement, this might represent a limited form of brachial plexopathy, in the context of a diffuse immune-mediated polyneuropathy, given the absence of brachial plexus injury at neck MR.

In conclusion, SS patients may be affected by cranial neuropathy, mainly optic neuritis or trigeminal neuropathy, which could represent the first symptom of subclinical connective tissue disease. In addition, the first observation of phrenic neuritis in a woman affected by SS and scleroderma in overlap was also described. Overall, careful clinical evaluation is recommendable in such conditions, particularly in individuals with apparently isolated cervicocranial nerve involvement.

## Figures and Tables

**Table 1:** Review of the literature regarding Sjögren's syndrome (SS) patients with cranial neuritis.

First authors/year	Ref.	No. pts.	Age/gender	Onset of neuritis versus diagnosis of SS (years)∗	Nerves involved/clinical features	Follow-up
Sjogren/1935	[10]	1	n.d.	n.a.	bil. VII	n.a.

Weber/1945	[11]	1	n.a.	n.a.	III	n.a.

Spillane/1959	[12]	1	58 F	n.a.	bil. V	n.a.

Attwood/1961	[13]	1	50 F	n.a.	III–V–VII–IX	Improv. with Cs

Kaltreider/1969	[14]	4	45 F, 48 F, 52 F, 73 F	+6; +2; +5; +14	sV (3), bil. (pt. 3); bil. I + PNS involv. (pt. 4)	1: chronic course, onset during Cs; 2, 4: Cs inefficacy; 3: response to Cs
		1	55 F	+8	I	remission within 1 year

Whaley/1972	[15]	1	27 F	n.a.	V	Response to Cs and P.E.

Hull/1984	[16]	1	33 F	0	V + PNS	n.a.

Vincent/1985	[17]	1	53 F	+7	V–VII–IX-diplopia	Recurrent ep. (6 in 7 years)

Alexander/1986	[18]	16	Mean 50 F	n.a.	7 II, 2 III, 1 V, 6 VI, 1 VII, 1 VIII	n.a.

Shimode/1986	[19]	1	n.a.	n.a.	II	n.a.

Serratrice/1986	[20]	1	58 F	+4	V	Cs 20 mg/day ineffective

Malinow/1986	[21]	1	F	n.a.	V + d.r. ganglionitis	n.a.

Mauch/1994	[22]	1	n.a.	n.a.	V	n.a.

Hankey/1987	[23]	1	78 F	+ (several years)	V + PNS involv.	n.a.

Wise/1988	[24]	3	F	− (1–6)	II	n.a.

Laloux/1988	[25]	1	81 F	n.a.	V + d.r. ganglionitis	n.a.

Graus/1988	[26]	2	58 F, 75 F	n.a.	sV	n.a.

Phanthumchinda/ 1989	[27]	1	28 F	0	Multiple	Resolved with Cs

Uchihara/1989	[28]	1	n.a.	n.a.	bil. VII	n.a.

Mellgren/1989	[29]	5	n.a.	n.a.	V	n.a.

Andonopoulos/ 1990	[30]	3	n.a.	n.a.	V	n.a.

Flint/1990	[31]	1	n.a.	n.a.	bil. sV	n.a.

Mukai/1990	[32]	5	n.a.	n.a.	V	n.a.

Semah/1990	[33]	1	57 F	−11	V	Chronic course

Berman/1990	[34]	1	10 F	0	II + CNS vasculitis	Improv. with immunosuppressors

Berault-Dupont/1992	[35]	1	59 F	0	VII	Improv. to high dosage Cs

Tesar/1992	[36]	3	20 F, 35 F, 41 F	0; −1; −2	II, bil. III-IV–VI (pt. 1), bil. II (pt. 2), II–IX-diplopia + CNS involv. (pt. 3)	Resolution with high dosage Cs (pt. 1); improv. with high dosage Cs (pt. 2); recurrent, resolution of neuritis with Cs/CYC within 6 months (pt. 3)

Soubrier/1993	[37]	4	n.a.	n.a.	V	n.a.

Pou Serradell/1993	[38]	1	63 F	− (n.a.)	III (8 ep.), multiple (5 ep.)	Recurrent, responsive to Cs

Güell/1993	[39]	1	n.a.	0	V	n.a.

Mauch/1987	[40]	1	n.a.	n.a.	sV	n.a.

Bakouche/1994	[41]	1	49 M	− (n.a.)	Diplopia-V-tinnitus	Recurrent (3 ep.) solving within 3 weeks

Matsukawa/1995	[42]	1	56 F	0	V then IV–VI	The first ep. autoresolved; the second with Cs

Harada/1995	[43]	1	n.a.	− (n.a.)	II + acute transv. myelopathy	Resistant to Cs

Tajima/1997	[44]	9	Mean 54.9 F; 51 F	n.a.	8 V, 4 bil.; 1 II	n.a.

Rojas-Rodriguez/1998	[45]	1	8 F	− (n.a.)	II	Visual impairment not responsive to Cs, IVIG, CYC pulses

Govoni/1999	[4]	2	51 F, 24 F	+6; +2	VIII; III + cerebellar ataxia	n.a.

Dumas/1999	[46]	1	41 F	n.a.	sV	n.a.

Kuhl/1999	[47]	1	54 F	+8	IV	Improv. spontaneously within few weeks

Touze/1999	[48]	1	34 F (first ep.)	−35; −2; 0; +2; + (n.a.)	VI, VII, IX, laryngeal	Recurrent ep., not improv. with Cs

Oketani/1999	[49]	1	52 F	+2	II	Improv. to pulse Cs

Wingerchuk/1999	[104]	4	n.a.	n.a.	II	n.a.

Chu/2000	[50]	1	54 F	0	IV-V	Resolution with Cs and Aza within 4 weeks

Urban/2001	[51]	1	53 F	+1	V–IX-X	Chronic course

Hadithi/2001	[52]	1	41 F	0	VII	Autoresolution after several days

Lafitte/2001	[53]	1	n.a.	n.a.	V	n.a.

Kadota/2002	[54]	1	63 F	0	II	Autoresolution within 6 months

Maeda/2002	[55]	1	21 F	0	II	Autoresolution

Anaya/2002	[56]	1	n.a.	n.a.	II	n.a.

Yanagihara/2002	[57]	1	39 F	0	III	n.a.

Font/2003	[58]	6	Mean 58 F	−4 to 0	V	Chronic course besides oral Cs

Delalande/2004	[59]	30	n.a.	n.a.	2 I, 13 II, 5 V, 4 VII, 6 VIII	n.a.

Mori/2005	[60]	20	Mean 55.6	− (n.a.)	15 V, 1 VII, 5 multiple (III, V, VI, VII, IX, X, XII)	4/7 patients improv. with Cs

Rousso/2005	[61]	1	40 F	0	VII	Recurrent ep. that autoresolved, in 1 pt. with Cs/vit. B12 within 12 days

Oishi/2007	[62]	1	65 M	0	VI	Autoresolved within 5 months

Cardoso/2006	[63]	1	9 F	−9; 0	bil. II	2 ep. 9 years apart, irreversible visual loss after initial response to Cs

Galbussera/2007	[64]	1	79 F	0	III	Resolution with Cs and IGIV within 2 months

Pournaras/2007	[65]	1	38 M	0	bil. II	Improv. with Cs

Gökçay/2007	[66]	1	20 F	−10	II	Cs/Aza resistant, switch to CYC

Arabshahi/2006	[67]	1	11 F	0	bil. II + transv. myelitis	Recurrent ep., transient improv. with Cs

Barroso/2007	[68]	1	34 F	−9	II + CNS involv.	Recurrent ep., responsive to iv Cs

Teo/2008	[69]	1	52 F	0	III	n.a.

Béjot/2008	[70]	1	53 F	+ (n.a.)	bil. II + aseptic meningitis	Improv. with CYC

Lui/2008	[71]	1	59 M	0	III	Resolved with Cs/Aza within 2 weeks

Ii/2008	[72]	1	49 F	0	II	Improv. with IVIG

Javed/2008	[73]	2	n.a.	n.a.	II	n.a.

Pittock/2008	[74]	6	n.a.	n.a.	II	n.a.

Min/2009	[75]	6	n.a.	n.a.	II	n.a.

Ashraf/2009	[76]	1	47 F	0	V–IX–XII	Resolved with Cs and MTX

Kato/2009	[77]	1	25 F	+ (2)	II + CNS involv.	Improv. with high-dosage Cs

Dellavance/2009	[78]	2	n.a.	n.a.	II	n.a.

Kim/2009	[79]	7	n.a.	n.a.	II	Poor prognosis for high relapse rate

Alhomoud/2009	[80]	10	Mean 40 F	n.a.	9 II + CNS involv.; 1 VII	n.a.

Rabadi/2010	[81]	1	23 F	0	II	n.a.

Sakai/2010	[82]	1	77 M	0	III-bil.V-VI-VII	Improv. with Cs

Imbe/2010	[83]	1	31 F	0	II + acute myelitis	Improv. with Cs/P.E.

Nascimento/2010	[84]	2	n.a.	−10; −0.5	V	n.a.

Chourkani/2010	[85]	2	43 F, 48 F	0	II (bil. 1 pt.)	Improv. with Cs/immunosuppressors

Massara/2010	[86]	3	48, 50, 74 F	+5, +6, +16 years later	2 II, 1 VII	Resolution with iv Cs

Cojocaru/2011	[87]	1	50 F	+0.7; +2	II then V	1° ep.: improv. with Cs/IVIG; 2° ep.: Cs-resistant

Niţescu/2011	[88]	2	n.a.	+ (n.a.)	II	n.a.

Yadav/2011	[89]	1	26 F	+ (several years)	bil. II	n.a.

Koga/2011	[90]	1	31 F	0	II + acute myelitis	Improv. with P.E., Cs-resistant

Gono/2011	[91]	7	Mean 44 F	n.a.	3 II (1 + CNS involv.), 2 V, 1 VII, 1 IX-X	n.a.

Kolfenbach/2011	[92]	6	n.a.	n.a.	II	Recurrent ep.

Estiasari/2012	[93]	10	n.a.	n.a.	II	n.a.

Horai/2012	[94]	8	63 F, 52 M, 43 F, 61 F, 51 F, 55 F, 45 F, 35 F	0 (first case); n.a.	V (4 bil.)	Improv. with Cs/tacrolimus (1); Improv. with Cs (1); Improv. with symptomatic treat (3); resist. to Cs (1); recurrent (1)

Tan/2012	[95]	1	56 F	0	bil. II	Permanent visual impairment

Maruta/2012	[96]	1	89 F	0	II	Improv. with iv Cs

Mallucci/2012	[97]	1	74 F	0	bil. II	n.a.

Teixeira/2013	[7]	4	Mean 47.9 F	n.a.	2 II, V, VI, 2 VII	Cs almost effective

Briani/2013	[98]	1	66 F	+5	V	n.a.

Flanagan/2013	[99]	1	64 M	0	V	n.a.

Tang/2013	[100]	8	Mean 34.7 F	0	II	Recurrent (3 pts), response to Cs/immunosuppressors

Present cases		2	40 F, 54 F	−6; +8	II; phrenic nerve	Improvement with Cs (40 F), with IVIG (54 F)

TOTAL		**267**	**F/M: 20.8 ** **Mean age: **48 ± 13.2** years**		**4 I, 123 II, 8 III, 4 IV, 95 V, 17 VI, 23 VII, 9 VIII, 11 IX, 8 X, 0 XI, 6 XII (**§**) **	

(∗) 0: diagnosis of SS and neuritis were contemporary; + (yrs): neuritis onset after diagnosis of SS; − (yrs): neuritis onset before diagnosis of SS. (§) for each cranial nerve, all cases of documented involvement have been counted, even if they are included in episodes of multineuritis; therefore the total of SS patients with neuritis does not correspond to the total number of episodes with cranial nerve involvements. n.a. = not available; CNS = central nervous system; PNS = peripheral nervous system; bil. = bilateral; involv. = involvement; sV = pure sensory trigeminal involvement; d.r. ganglionitis = dorsal roots ganglionitis; transv. = transverse; ep. = episode; pt. = patient; Cs = corticosteroids; CYC = cyclophosphamide; Aza = azathioprine; IMTX = methotrexate; VIG = intravenous immunoglobulins; P.E. = plasma exchange.

**Table 2:** Synoptic view of cranial nerve involvement in the course of Sjögren's syndrome (SS).

Cranial nerve(s)	Frequency of involvement (%)	Key points
I	1	Probably underdiagnosed because of the concomitant olfactory dysfunction secondary to mucosal dryness.

II	46	Possible feature of neuromyelitis optica spectrum disorder, associated with SS, otherwise, autoimmune neuritis in patients with possibly misdiagnosed SS.

III, IV, VI	12	Clinically evident as diplopia, this neuritis is generally responsive to steroids.

V	38	Autoimmune damage of the Gasser ganglion could be suspected. Neuritis tends to be recurrent or to stabilize and to be less frequently responsive to steroids than other cranial neuritis.

VII	5	Neuritis isolated or associated with another nerve involvement, with good prognosis.

VIII	3	Neuritis rarely reported as tinnitus, often isolated.

IX, X, XI, XII	4	Transient, often recurrent neuritis episodes, generally responsive to steroids. The IX nerve was always reported in cases of multineuritis, while the XI was never mentioned in literature.

Cranial nerves are named with their Roman numerals. The frequencies of involvement refer to the cases illustrated in Table 1, considering a total of 267 SS patients with cranial neuritis.
